# Supplementary material for: Induced photoelectron circular dichroism onto an achiral chromophore
Source: Nat Commun. 2023 Oct 9;14:6290. doi: 10.1038/s41467-023-42002-1 (PMC10562374; doi:10.1038/s41467-023-42002-1)
Supplement: Supplementary file 1 — Supplementary Information [file 41467_2023_42002_MOESM1_ESM.pdf]

Induced Photoelectron Circular Dichroism onto an Achiral Chromophore

Etienne Rouquet, [1,2] *Madhusree Roy Chowdhury,* [1] *Gustavo A. Garcia,* [1] *Laurent Nahon* [1] *

Jennifer Dupont, [2] *Valéria Lepère,* [2] *Katia Le Barbu-Debus* [2] *Anne Zehnacker* [2] *

1) Synchrotron SOLEIL, L'Orme des Merisiers, Départementale 128, F-91190 St. Aubin, France

2) Institut des Sciences Moléculaires d'Orsay (ISMO), CNRS, Université Paris-Saclay, F-91405 Orsay, France

Corresponding Authors

*Laurent Nahon laurent.nahon@synchrotron-soleil.fr *Anne Zehnacker anne.zehnacker-rentien@universite-paris-saclay.fr

SUPPLEMENTARY INFORMATION

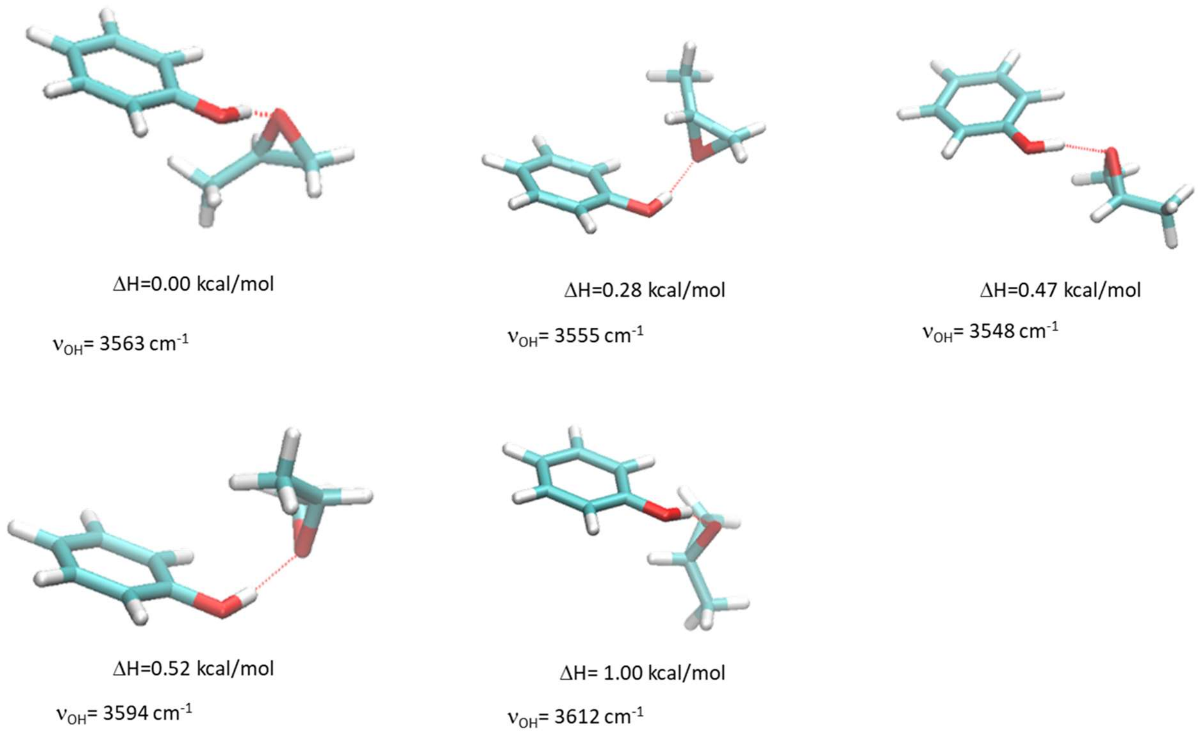

Supplementary Figure 1 │ Structure of the phenol (*S*) methyloxirane complex. Most stable calculated structures of the Phe:MOx complex, and corresponding OH stretching mode frequencies calculated at the B3LYP-D3BJ/6-311++G(d,p) level of theory. The hydrogen bond is indicated by a dotted line. The calculated harmonic frequencies are scaled by 0.953. The free energy ΔH relative to the most stable complex is given in kcal/mol.

	HOMO	HOMO-1	HOMO-2	Structures
a) Phe:MOx	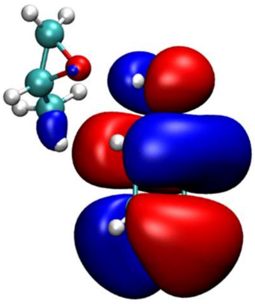 IE = 8.11 eV	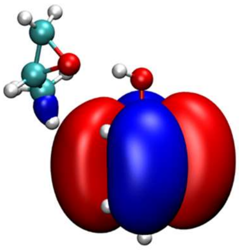 IE = 8.93 eV	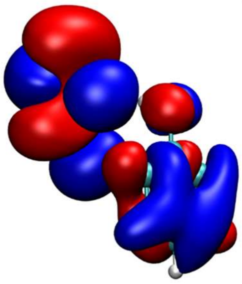 IE = 11.48 eV	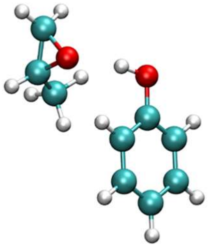
b) Phe	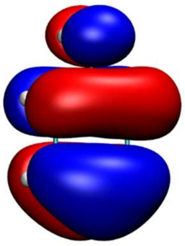 IE = 8.53 eV	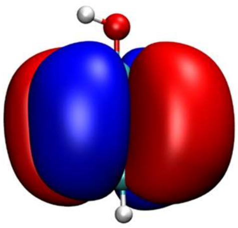 IE = 9.28 eV	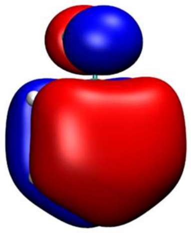 IE = 11.79 eV	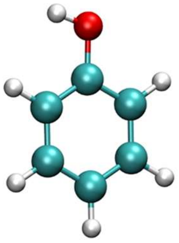
c) MOx	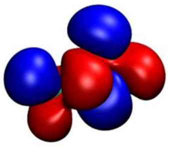 IE = 10.72 eV	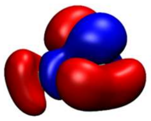 IE = 11.09 eV	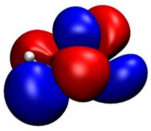 IE = 13.18 eV	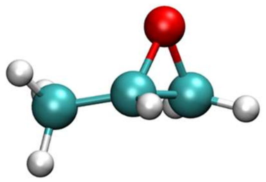

Supplementary Figure 2│ Localisation of the frontier orbitals Frontiers orbitals calculated for the Phe:MOx complex as well as bare phenol and bare methyloxirane at the MP2/6-31++G(d,p) level. The electronic density was plotted with an isodensity value of 0.01. Calculated vertical ionisation energies (IE) are obtained with the outer valence Green's function (OVGF) method and cc-pVTZ basis set.

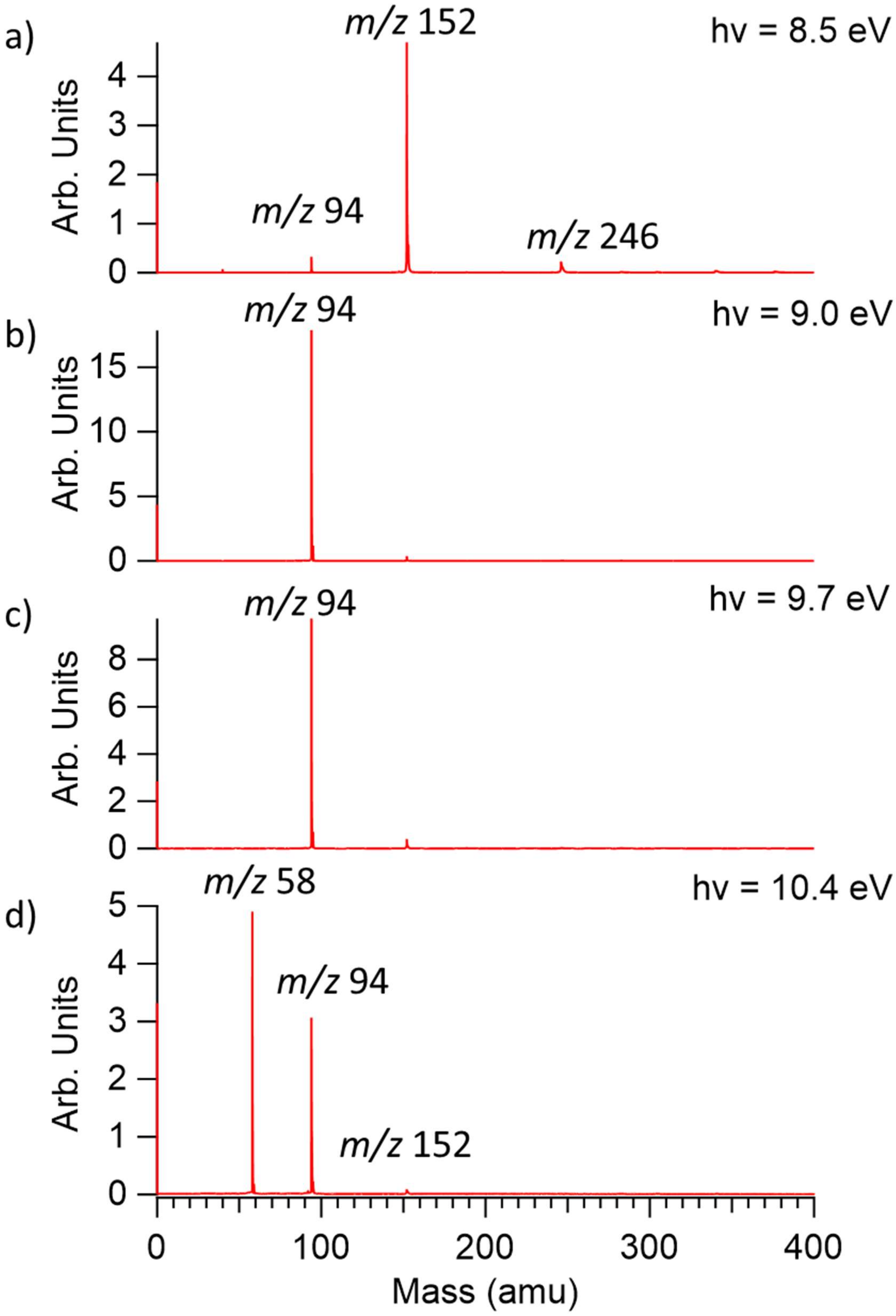

Supplementary Figure 3 │ Mass spectra. Mass spectra recorded at photon energies of 8.5, 9.0, 9.7 and 10.4 eV. The peaks at *m/z* 58, 94, 152, and 246 correspond to MOx, Phe, Phe:MOx, and Phe_2:MOx, respectively.

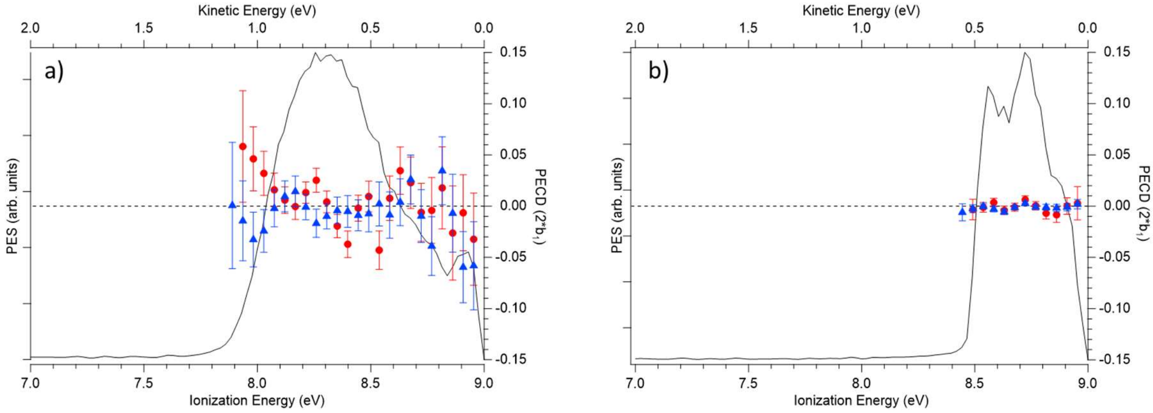

Supplementary Figure 4: │ **Photoelectron spectra and dichroic parameter at 9 eV for the complex of phenol with the two enantiomers of methyloxirane** a) Photoelectron spectra (PES - full black line) and photoelectron circular dichroism (PECD) obtained for the complex of phenol with (*S*) MOx (red circles)) and (*R*) MOx (blue triangles), recorded in coincidence with the mass *m/z* 152 at a photon energy of 9.0 eV. b) Same experimental data recorded at the mass of bare phenol (*m/z* 94).

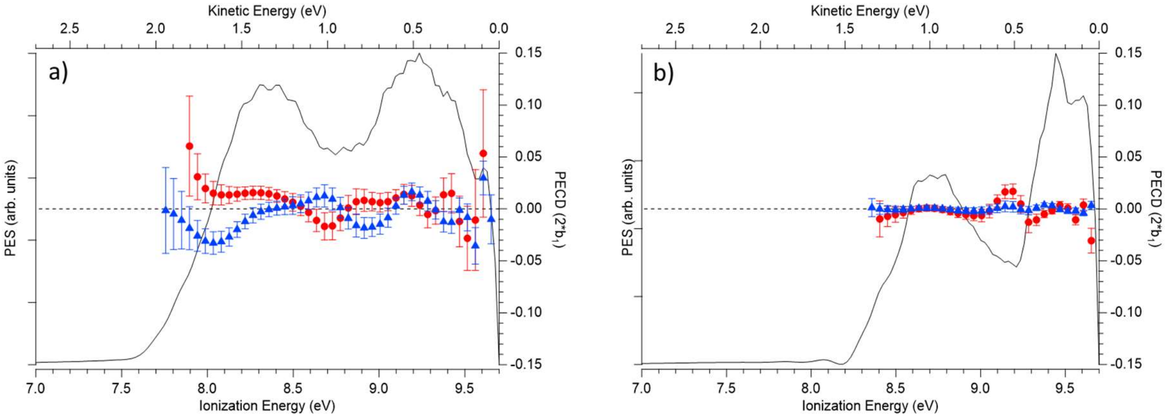

Supplementary Figure 5 │ **Photoelectron spectra and dichroic parameter at 9.7 eV for the complex of phenol with the two enantiomers of methyloxirane** a) Photoelectron spectra (PES - full black line) and photoelectron circular dichroism (PECD) obtained for the complex of phenol with (*S*) MOx (red circles) and (*R*) MOx (blue triangles), recorded in coincidence with the mass *m/z* 152 at a photon energy of 9.7 eV. b) Same experimental data recorded at the mass of bare phenol (*m/z* 94).

Study of the Phe_2:MOx complex

Due to the absence of signal in REMPI experiments, IR-UV double resonance experiments on the Phe_2:MOx complex were not possible. Instead, the most stable structures were calculated following the same procedure as described for the 1:1 complex. The two most stable structures, shown in Supplementary Figure 5, consist in a phenol dimer hydrogen-bonded to the MOx molecule. Despite the fact that the phenol sub-unit acting as a donor in the phenol dimer is not directly hydrogen-bound to the MOx molecule, the distance from the centre of its aromatic ring to the latter is only 4.8 Å. Moreover, a weak CH…π interaction takes place with a distance between CH and the centre of its aromatic ring of 2.9 Å. The structure of the phenol dimer within the Phe_2:MOx complex is very close to that of the isolated phenol dimer, as determined by high-resolution rotational spectroscopy.[1] As already mentioned for the Phe:MOx complex, the two lone pairs of the MOx are not equivalent due to the methyl substituent, which results in two almost isoenergetic complexes of the Phe_2:MOx complex.

The photoelectron spectrum, recorded by monitoring the electrons in coincidence with the ions at the mass of the Phe_2:MOx complex (*m/z* 246) is shown in Supplementary Figure 6, using a photon energy of 8.5 eV. The ionisation energy threshold is identical for the Phe_2:MOx and Phe:MOx complexes. The PES of Phe_2:MOx slightly differs from that of Phe:MOx, with a first maximum at 8.1 eV. This slightly different shape results from the presence of four close-lying orbitals, namely, two π orbitals on each phenol ring. The lower-energy orbitals are shown in Supplementary Figure 6. The HOMO and HOMO-2 of the Phe_2:MOx complex correspond to the phenol HOMO and HOMO-1 located on the ring in direct interaction with the MOx, while the HOMO-1 and HOMO-3 correspond to the phenol HOMO-1 located on the ring that is further from the MOx. A clear PECD asymmetry is evidenced for electron kinetic energies below 0.5 eV, where the HOMO-1 and HOMO-2 are expected to be ionised. This result indicates that both phenol rings are sensitive to the chirality of the MOx host, even the phenol not directly hydrogen-bound to it but interacting through a much weaker CH…π interaction.

	HOMO	HOMO-1	HOMO-2	HOMO-3	HOMO-4	HOMO-5	Structure
Phe_2-MOx (1)	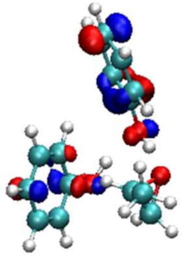 8.34 eV	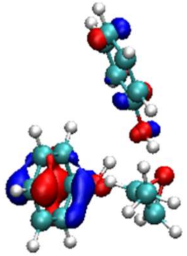 8.52 eV	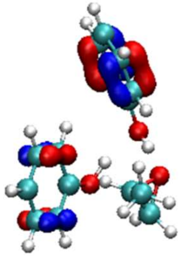 9.14 eV	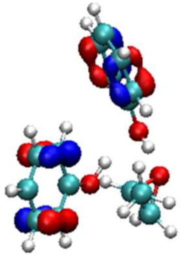 9.29 eV	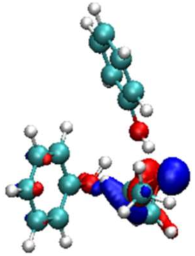 11.28 eV	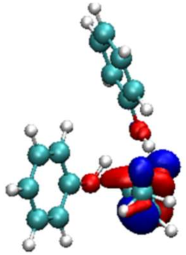 11.43 eV	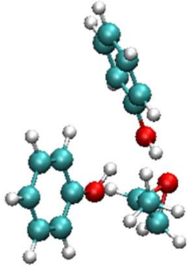 ΔH=0
Phe_2-MOx (2)	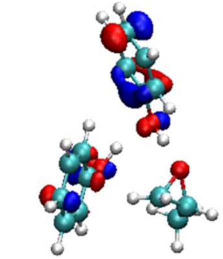 8.40 eV	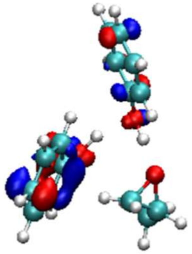 8.48 eV	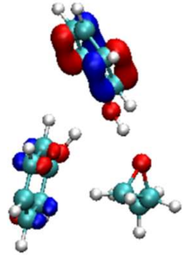 9.13 eV	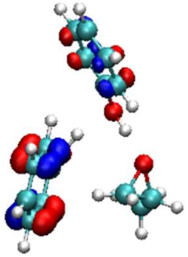 9.25 eV	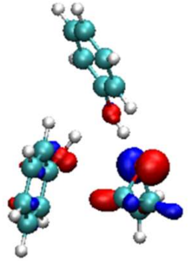 11.21 eV	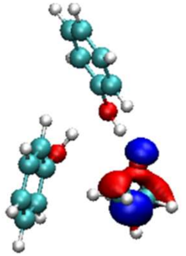 11.48 eV	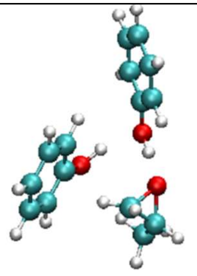 ΔH=0.15 kcal/mol

Supplementary Figure 6 │ Localisation of the frontier orbitals Calculated frontiers orbitals for the most stable structures of the Phe_2:MOx complex at the MP2/6-31++G(d,p) level. The electronic density was plotted with an isodensity value of 0.07. Calculated vertical ionisation energies (IE) are given in eV and are obtained with the outer valence Green's function (OVGF) method and cc-pVTZ basis set.

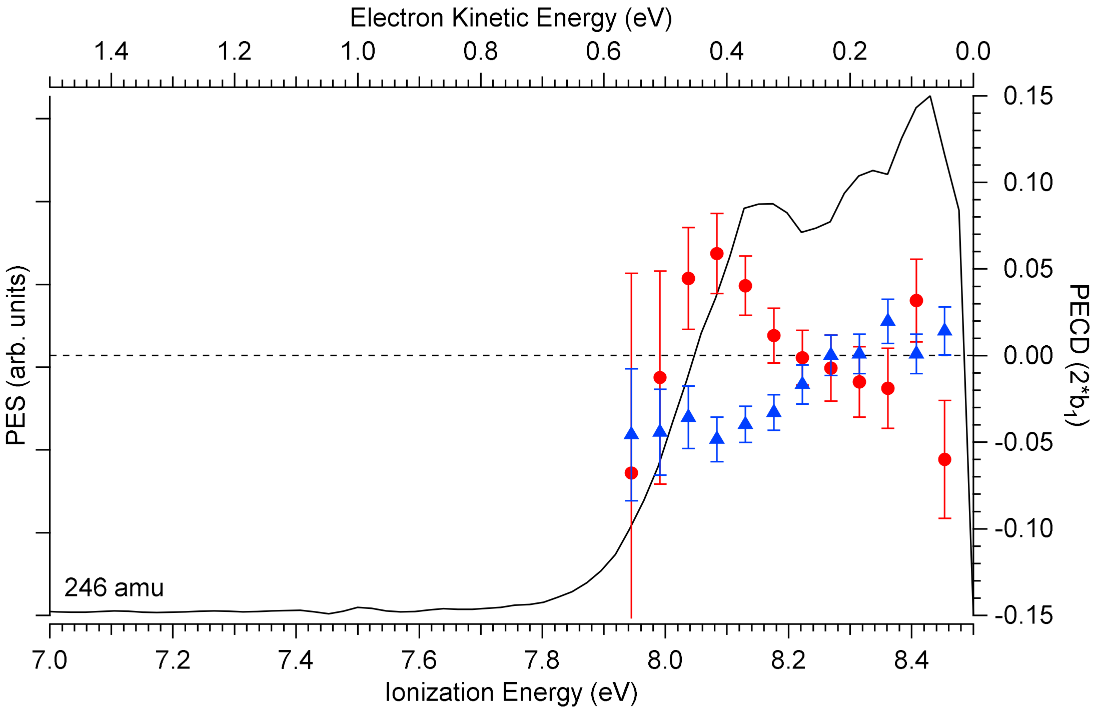

Supplementary Figure 7 | Photoelectron spectra and dichroic parameter at 8.5 eV for the complex made of two phenol molecules and the two enantiomers of methyloxirane a) Photoelectron spectra (PES- full black line) and photoelectron circular dichroism (PECD) obtained for the trimeric complex composed of two phenol molecules and (*S*) MOx (red circles) and (*R*) MOx (blue triangles), recorded in coincidence with the mass *m/z* 246 at a photon energy of 8.5 eV.

Supplementary References

1. Seifert NA, et al. The interplay of hydrogen bonding and dispersion in phenol dimer and trimer: structures from broadband rotational spectroscopy. Physical Chemistry Chemical Physics 15, 11468-11477 (2013).
